# Early Neurodegeneration Progresses Independently of Microglial Activation by Heparan Sulfate in the Brain of Mucopolysaccharidosis IIIB Mice

**DOI:** 10.1371/journal.pone.0002296

**Published:** 2008-05-28

**Authors:** Jérôme Ausseil, Nathalie Desmaris, Stéphanie Bigou, Ruben Attali, Sébastien Corbineau, Sandrine Vitry, Mathieu Parent, David Cheillan, Maria Fuller, Irène Maire, Marie-Thérèse Vanier, Jean-Michel Heard

**Affiliations:** 1 Unité Rétrovirus et Transfert Génétique, INSERM U622, Department of Neuroscience, Institut Pasteur, Paris, France; 2 Service de Neurologie Pédiatrique, Hôpital Bicêtre, Assistance Publique/Hôpitaux de Paris, INSERM U802, 94000, le Kremlin-Bicêtre, France; 3 Groupement hospitalier est, CBPE, Bron, France; 4 Genetic Medicine, Children, Youth and Women's Health Service, North Adelaïde, Australia; 5 INSERM U820, Faculté de Médecine Laënnec, Lyon, France; Massachusetts General Hospital and Harvard Medical School, United States of America

## Abstract

**Background:**

In mucopolysaccharidosis type IIIB, a lysosomal storage disease causing early onset mental retardation in children, the production of abnormal oligosaccharidic fragments of heparan sulfate is associated with severe neuropathology and chronic brain inflammation. We addressed causative links between the biochemical, pathological and inflammatory disorders in a mouse model of this disease.

**Methodology/Principal Findings:**

In cell culture, heparan sulfate oligosaccharides activated microglial cells by signaling through the Toll-like receptor 4 and the adaptor protein MyD88. CD11b positive microglial cells and three-fold increased expression of mRNAs coding for the chemokine MIP1α were observed at 10 days in the brain cortex of MPSIIIB mice, but not in MPSIIIB mice deleted for the expression of Toll-like receptor 4 or the adaptor protein MyD88, indicating early priming of microglial cells by heparan sulfate oligosaccharides in the MPSIIIB mouse brain. Whereas the onset of brain inflammation was delayed for several months in doubly mutant versus MPSIIIB mice, the onset of disease markers expression was unchanged, indicating similar progression of the neurodegenerative process in the absence of microglial cell priming by heparan sulfate oligosaccharides. In contrast to younger mice, inflammation in aged MPSIIIB mice was not affected by TLR4/MyD88 deficiency.

**Conclusions/Significance:**

These results indicate priming of microglia by HS oligosaccharides through the TLR4/MyD88 pathway. Although intrinsic to the disease, this phenomenon is not a major determinant of the neurodegenerative process. Inflammation may still contribute to neurodegeneration in late stages of the disease, albeit independent of TLR4/MyD88. The results support the view that neurodegeneration is primarily cell autonomous in this pediatric disease.

## Introduction

Divergent events such as deposition of Aß in Alzheimer disease [1], α-synuclein or neuromelanin in Parkinson disease [2], [3], disease-associated PrP^sc^ protein in prion disease [4], mutants SOD1 protein in amyotrophic lateral sclerosis [5], viral proteins in human immunodeficiency infection [6] or mutant huntingtin in Huntington disease [7] initiate involvement of the immune system, which in turn interacts with the nervous system and set the pace of progressive neurodegeneration. Neurodegenerative diseases are characterized by both local activation of resident microglia and astrocytes and infiltration of leucocytes from the periphery. Immune reaction can be toxic for neurons through the local production of the inflammatory cytokines TNFα, IL-1 and IL-6 [8], [9]. Inflammatory chemokines, especially the macrophage inflammatory protein 1α (MIP1α), are also produced, stimulating microglial activation and attracting peripheral inflammatory cells to the brain parenchyma. Suppressing inflammation is regarded as a potential therapeutic approach against the development of neurodegeneration in AD and PD, especially with regard to the relative protection conferred by the long-term use of non-steroidal anti-inflammatory drugs [10], [11].

Whereas the association of chronic neurodegeneration and inflammation is well established, the causative links between these events is debated. Series of evidence from studies performed in animal models of chronic neurodegeneration suggest that microglial activation might be primed by the ongoing pathology rather than the opposite. The deletions of cytokine genes have shown inconsistent, minor or no effect on disease progression in mouse models of neurodegenerative disorders [12], [13]. It is moreover unclear how and to which extent inflammation alone could account for events almost invariably associated with neurodegeneration, such as mitochondrial dysfunction [14], altered axonal transport [15], altered calcium storage and endoplasmic reticulum functions [16], or dysfunction of intracellular protein degradation and macroautophagy pathways [17], [18].

Here, we addressed causative links between abnormal metabolite accumulation, brain inflammation and disease marker expression in a mouse model of pediatric neurodegeneration accompanying lysosomal storage diseases. Brain inflammation was documented in several of these diseases [19]–[25]. In mucopolysaccharidosis type III (MPSIII), the unique event responsible for both cell pathology and brain inflammation is the production and accumulation of partially digested, possibly abnormally sulfated or acetylated oligosaccharide fragments of heparan sulfate (HS), a specific type of glycosaminoglycan (GAG). Interruption of HS oligosaccharide degradation is the consequence of a defect in one of the four exoglycanases required for the removal of the α-linked N-acetylglucosamine at the non-reducing end of the saccharide chain. The production of HS oligosaccharides is associated to the secondary accumulation of GM2 and GM3 gangliosides [26], [27], to the formation of large cytoplasmic inclusions in various brain cell types [28], to the accumulation of subunit C of the mitochondrial ATP synthase [29], and to the dysregulation of GAP43 mRNA expression in brain tissue [30]. Early onset neurological manifestations in children lead to severe progressive mental retardation and premature death. Our gene therapy studies in the mouse [31] and the dog (M. Ellinwood, J.M. Heard, unpublished) models of MPSIII subtype B (MPSIIIB), a deficiency of α-N-acetylglucosaminidase (NaGlu), showed that delivery of the missing enzyme to the brain prevented pathology and clinical manifestations. Here, we provide evidence that microglial cell activation is primed by HS oligosaccharides at very early stage of the disease, although suppression of microglial cell priming by HS oligosaccharides does not modify the course of the neurodegenerative process.

## Results

### HS oligosaccharides activate microglial cells

**Figure 1:**
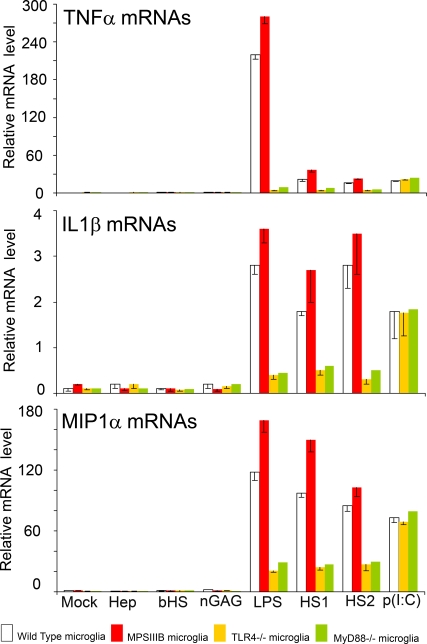
Heparan sulfate oligosaccharides activate mouse microglial cells in vitro. Microglial cell cultures were established from wild type (white bars), MPSIIIB (red bars), TLR4^−/−^ (yellow bars), or MyD88^−/−^ (green bars) mice. Cells were incubated for 4 hours with normal medium (mock), 5 µg/mL heparin (Hep), 5 µg/mL bovine heparan sulfate (bHS), 5 µg/mL of GAG purified from normal individual urine (nGAG), 1 µg/mL LPS (LPS), 5 µg/mL of heparan sulfate purified from the urines of two MPSIIIB patients (HS1 and HS2) or 10 µg/ml of polyinosine-polycytidylic acid (p[I∶C]). Normal mouse microglial activation is shown by increased amounts of TNFα, IL-1β, MIP1α mRNA production following incubation with LPS, HS1 or HS2. More robust responses of cells isolated from MPSIIIB mice is consistent with previous in vivo microglial priming, possibly by heparan sulfate. Lower expression by microglia from TLR4^−/−^ or MyD88^−/−^ mice indicates signaling through these molecules, whereas activation persisted upon stimulation by p[I∶C], which binds TLR3. Total RNA was extracted, reverse transcribed and amounts of cDNAs coding for TNFα, IL-1ß, MIP1α, or the reference protein ARPO [53] were measured by Q-PCR. Indicated values are means±SEM of ratios of TNFα, IL-1ß, MIP1α mRNAs to ARPO mRNAs measured in three independent experiments.

We examined whether microglial cell activation could be induced by HS oligosaccharides. For that purpose, HS oligosaccharides were purified from the urines of two MPSIIIB patients (HS1 and HS2, see materials and methods and figure S1). Tandem mass spectrometry analysis identified HS oligosaccharide species previously detected in MPSIIIB patient urines [32] and in the brain of MPSIIIB dogs (M. Fuller, M. Elinwood and J. Hopwood, unpublished). It is therefore presumable that these molecules also accumulate in the brain of MPSIIIB mice. Normal mouse microglia cultures incubated with purified HS oligosaccharides released TNFα in culture supernatant (table 1) and showed morphological changes typical of cell activation (figure S2). This phenotype was similar to that induced by LPS. Morphological changes and release of TNFα in response to the stimulation by HS oligosaccharides were however not suppressed by polymixin B, a reagent that specifically inhibits LPS and other endotoxin action (table 1). Microglial cell activation induced by HS1 or HS2 led to increased detection of TNFα, IL1ß and MIP1α mRNAs (figure 1). Compared to LPS, HS oligosaccharides induced lower expression of TNFα mRNAs but equivalent levels of IL1ß and MIP1α mRNAs. Synthetic heparin, purified bovine HS and GAGs purified from normal individual urine did not activate microglial cultures (table 1, fig. 1 and figure S2). Microglial cell activation was much less intense when microglial cell cultures were derived from mice deficient for the expression of the innate immune response receptor Toll Like Receptor (TLR) 4 or the TLR adaptor protein MyD88 (figure 1). These results indicate that HS oligosaccharides produced by NaGlu deficient organisms stimulated microglial cell activation through the TLR4/MyD88 signaling pathway. Interestingly, higher amounts of IL1ß and MIP1α mRNAs were produced by microglial cells isolated from MPSIIIB mice in response to both LPS and HS oligosaccharides compared to wild type mouse microglia, suggesting higher susceptibility to innate immune stimulation (figure 1).

**Table 1:** Activated microglial cells release TNFα in culture supernatant.

Inducer	Concentration	TNFα (pg/ml)° Without PMB*	TNFα (pg/ml)° with PMB*(10 µg/ml)
None		<10	ND
Desulfated Heparin	5 µg/ml	<10	ND
Bovine HS	5 µg/ml	<10	ND
Normal GAG	5 µg/ml	<10	ND
LPS	0.5 µg/ml	125±12#	<10
LPS	1 µg/ml	581±52	<10
LPS	5 µg/ml	860±39	<10
HS2	0.5 µg/ml	<10	<10
HS2	1 µg/ml	12±1	12±1
HS2	5 µg/ml	26±5	28±5
HS1	5 µg/ml	59±7	45±6

° TNFα was detected in culture supernatants by ELISA (see materials and methods)

* PMB: polymyxin B, a drug that competes with endotoxin binding to TLR4. Absence of inhibition in the presence of PMB suggests that microglial activation was not induced by endotoxins.

# Values are means±SEM from 3 experiments, in which 3 microglial cell cultures were established from different mice.

**Figure 2:**
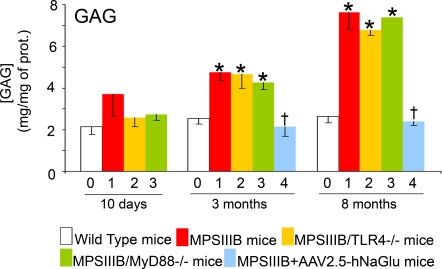
GAG accumulate in the brain of MPSIIIB mice. Wild type mice (0, white bars), MPSIIIB mice (1, red bars), MPSIIIB×TLR4^−/−^ mice (2, yellow bars), MPSIIIB×MyD88^−/−^ mice (3, green bars), or MPSIIIB mice in which the genetic defect was corrected in the brain by a single intracerebral injection of AAV2.5-hNaGlu vector (4, blue bars) were analyzed at the age of 10 days, 3 months, or 8 months. GAG concentration was determined in cortical tissue extracts. Values are means±SEM. Asterisks indicate significant difference with wild type mouse values, crosses indicate significant difference with MPSIIIB values (p<0.05, Mann and Whitney test).

With the aim to examine relationships between microglial cell priming by HS oligosaccharides and the progression of neuroinflammation and neurodegeneration in the brain with age, we studied MPSIIIB mice and produced MPSIIIB mice deficient for the expression of TLR4 or MyD88. As expected, MPSIIIB mice and doubly mutant MPSIIIB×TLR4^−/−^ or MPSIIIB×MyD88^−/−^ mice accumulated equivalent amounts of HS oligosaccharides in the brain, as appreciated by measuring GAGs in tissue extracts (figure 2).

### Priming of microglial cells is already prominent in the brain of 10-days-old MPSIIIB mice

**Figure 3:**
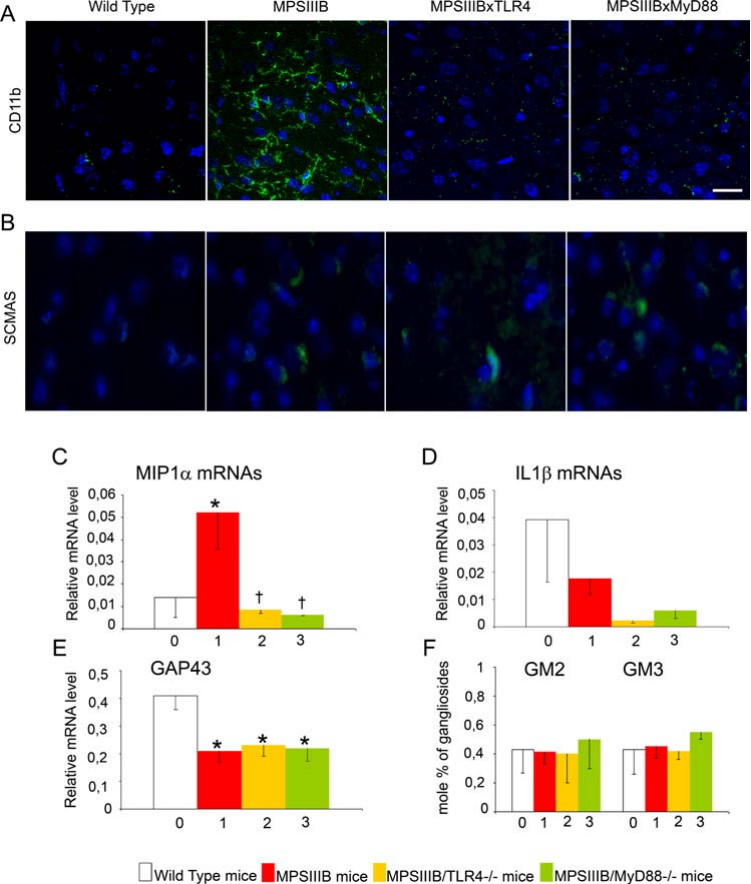
Microglial cell activation and pathology markers in the brain at 10 days. Wild type mice (0, white bars), MPSIIIB mice (1, red bars), MPSIIIB×TLR4^−/−^ mice (2, yellow bars), MPSIIIB×MyD88^−/−^ mice (3, green bars) were analyzed at the age of 10 days. Inflammation markers were studied in cortical samples stained with anti-CD11b antibody (green in A) and by measuring the relative amounts of MIP1α (C) and IL1ß (D) mRNAs by quantitative RT-PCR. Disease markers were studied in cortical samples stained with the anti-ScMAS antibody (green in B), and by measuring the relative amounts of GAP43 mRNAs (E) and the accumulation of GM2/GM3 gangliosides (F). Immunofluorescence (A and B): nuclei are stained in blue with Hoescht, scale bars: 20 µm for CD11b, 50 µm for ScMAS. Representative pictures from 3 MPSIIIB, 3 MPSIIIB×TLR4^−/−^ and 3 MPSIIIB×MyD88^−/−^ examined mice. RT-Q-PCR (C, D, E): mRNA amounts are expressed relative to the reference ARPO mRNA [53]. Asterisks indicate significant difference with wild type mice and crosses indicate significant differences with untreated MPSIIIB mice (p<0.05, Mann and Whitney non-parametric test). Number of mice used for GAG, gangliosides and mRNA analyses: wild type mice, n = 6; MPSIIIB mice, n = 6; MPSIIIB×TLR4^−/−^ mice, n = 5; MPSIIIB×MyD88^−/−^ mice, n = 2.

Brain cortical sections were immunostained for CD11b, a marker of microglial cells (figure 3A). Expression of CD11b was more intense in 10-days-old MPSIIIB mice than in age-matched wild type mice. Positive cells showed ramified morphology. IL1ß mRNAs were not expressed at significant level in 10-days-old MPSIIIB mice (figure 3D). However, MIP1α mRNA levels were already 3-folds higher than in wild type mice (figure 3C). Increased CD11b staining and MIP1α mRNAs expression suggested early priming of microglia in the brain of MPSIIIB. In doubly mutant mice, CD11b expression was lower than in MPSIIIB mice and equivalent to wild type mice. Increased MIP1α mRNA expression was not observed in the brain. These results suggest that increased CD11b and MIP1α mRNA expression in MPSIIIB mice depended on TLR4 and MyD88. Since HS oligosaccharides are already produced in NaGlu deficient mice at 10 days, this result is consistent with priming of microglial cells by HS oligosaccharides in 10-days-old MPSIIIB mice.

Mouse brain cortices were examined for altered expression of disease markers (figure 3B and C–F), including GM2 and GM3 ganglioside accumulation, cell vacuolation (not shown), ScMAS immunostaining and GAP43 mRNA expression level. MPSIIIB mice showed reduced expression of GAP43 mRNAs and a mild increased of ScMAS reactivity compared to age-matched wild type mice. Similar reduction of GAP43 mRNA detection and augmentation of ScMAS staining were observed in cortical extracts from doubly mutant mice, indicating that these alterations could take place in the absence of microglial cell priming by HS oligosaccharides.

### Inflammation was reduced but pathology was severe in doubly mutant mice at 3 months

**Figure 4:**
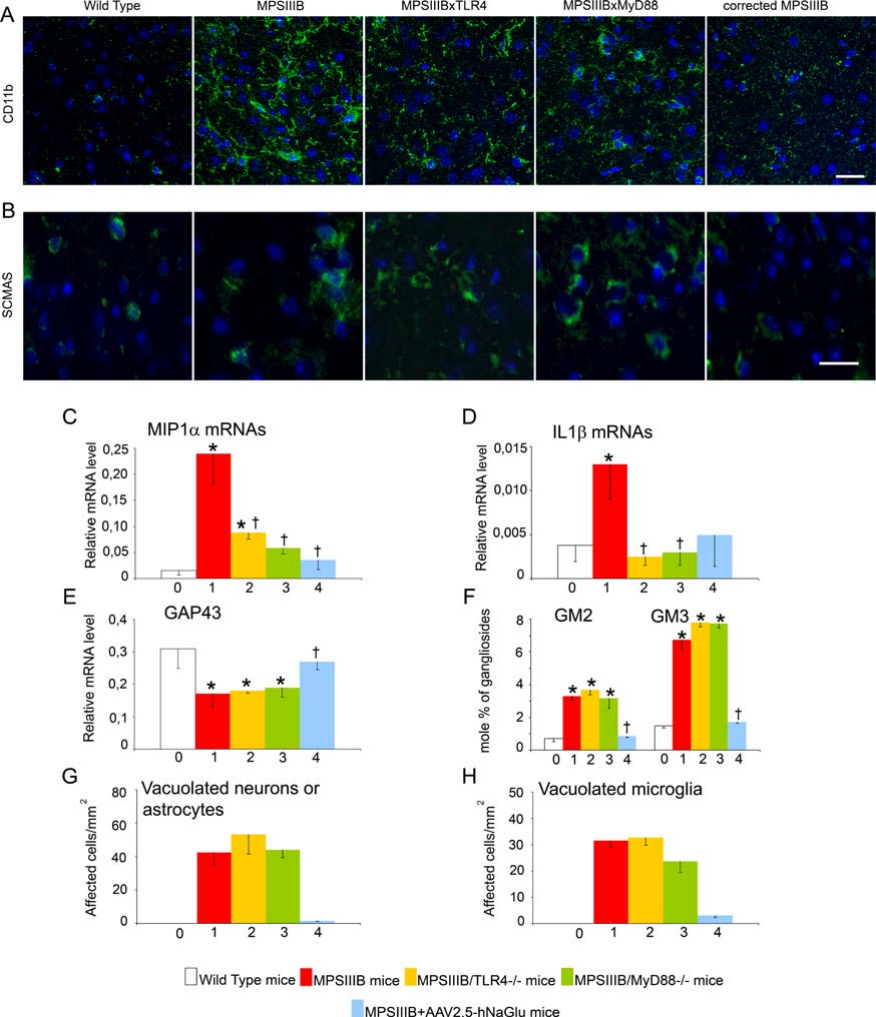
Microglial cell activation and pathology markers in the brain at 3 months. Wild type mice (0, white bars), MPSIIIB mice (1, red bars), MPSIIIB×TLR4^−/−^ mice (2, yellow bars), MPSIIIB×MyD88^−/−^ mice (3, green bars), or MPSIIIB mice in which the genetic defect was corrected in the brain by a single intracerebral injection of AAV2.5-hNaGlu vector (4, blue bars) were analyzed at the age of 3 months. Inflammation markers were studied in cortical samples stained with anti-CD11b antibody (green in A) and by measuring the relative amounts of MIP1α (C) and IL1ß (D) mRNAs by quantitative RT-PCR. Disease markers were studied in cortical samples stained with the anti-ScMAS antibody (green in B), and by measuring the relative amounts of GAP43 mRNAs (E), the accumulation of GM2/GM3 gangliosides (F), and the frequencies of vacuolated neurons or astrocytes (G) and vacuolated microglia in cortical semi-thin sections (H). Immunofluorescence (A and B): nuclei are stained in blue with Hoescht, scale bars: 20 µm for CD11b, 50 µm for ScMAS. Representative pictures from 3 MPSIIIB, 3 MPSIIIB×TLR4^−/−^ and 3 MPSIIIB×MyD88^−/−^ mice. RT-Q-PCR (C, D, E): mRNA amounts are expressed relative to the reference ARPO mRNA [53]. Pathology (G and H): semi-thin sections (1 µm) were stained with toluidin blue. At least 90 neurons/astrocytes were scored per mm^2^ section surface. Values are from 3 MPSIIIB, 3 MPSIIIB×TLR4^−/−^ and 3 MPSIIIB×MyD88^−/−^ examined mice. Examples of the morphology of cells that were scored as normal neurons or astrocytes, or vacuolated neurons or astrocytes are illustrated in figure S4. Asterisks indicate significant difference with wild type mice and crosses indicate significant differences with untreated MPSIIIB mice (p<0.05, Mann and Whitney non-parametric test). Number of mice used for GAG, gangliosides and mRNA analyses: wild type mice, n = 7; MPSIIIB mice, n = 10; MPSIIIB×TLR4^−/−^ mice, n = 3; MPSIIIB×MyD88^−/−^ mice, n = 3; MPSIIIB+AAV2.5-hNaGlu mice, n = 3.

CD11b staining was dramatically increased in the brain of MPSIIIB mice at 3 months, compared to age-matched wild type mice (figure 4A). Staining was also more visible in doubly mutant mice than in wild type mice, though much less intense than in MPSIIIB mice. In all cases, CD11b positive cells conserved their ramified morphology. IL1ß and MIP1α mRNAs were dramatically increased in 3-months-old MPSIIIB mice (figure 4C, D). In contrast, doubly mutant mice showed normal levels of IL1ß mRNAs and normal or slightly increased levels of MIP1α mRNAs. These results show that the development of inflammation in the brain of 3-months-old MPSIIIB mice was primarily the consequence of microglial cell priming involving TLR4 and MyD88.

To further document the role of HS oligosaccharide in this response, we treated MPSIIIB mice by gene therapy at the age of 6 weeks. Delivery of the missing enzyme NaGlu in the brain of MSIIIB mice normalized GAG levels in brain extracts (figure 2). CD11b staining in cortical sections was much less intense than in untreated mice, though slightly above levels detected in wild type mice (figure 4A). Detected IL1ß mRNA amounts were also slightly above wild type mouse level, though difference was not significant. MIP1α mRNA levels were equivalent to wild type mice (figure 4C,D). These results show that the alteration of inflammation markers in MPSIIIB mice was secondary to GAG accumulation, very low residual expression in treated mice being consistent with very low residual production of HS oligosaccharides, as expected since the therapeutic enzyme is not delivered to all brain cells [31].

All disease markers were affected in MPSIIIB mice at 3 months (figure 4B and E–H). GM2 and GM3 ganglioside levels were higher than in age-matched wild type mice. Large cytoplasmic inclusions were visible in various cell types including neurons, astrocytes, microglia, endothelial, epithelial and meningeal cells (examples are shown in figure S4). Immunostaining for ScMAS was increased. GAP43 mRNA levels were low compared to wild type, as previously shown [30]. Enzyme delivery by gene therapy and prevention of GAG accumulation was associated with a complete normalization of disease markers (figure 4B and E–H), indicating that toxic effects on brain cells were secondary to HS oligosaccharide accumulation. Disease markers were similarly detected in MPSIIIB and doubly mutant mice (figure 4B and E–H), indicating that neurodegeneration was not delayed when inflammation markers were absent or expressed at low level. Therefore, disease development at 3 months was mostly independent of microglial cell priming through TLR4 and MyD88.

### Inflammation and pathology were severe in MPSIIIB and doubly mutant mice at 8 months

**Figure 5:**
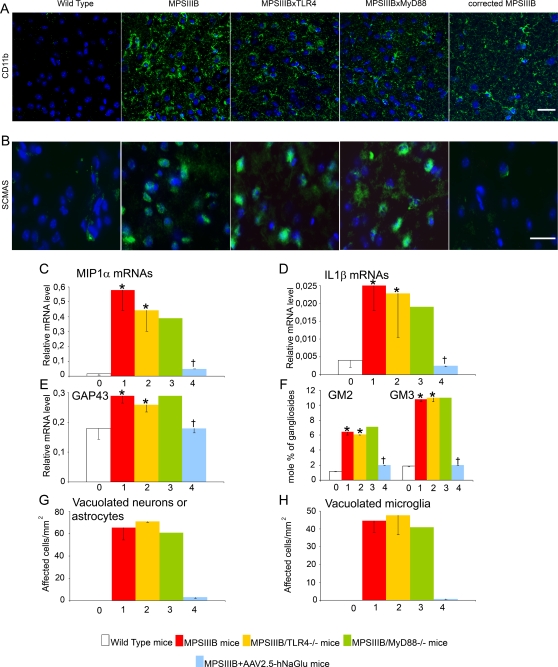
Microglial cell activation and pathology markers in the brain at 8 months. Wild type mice (0, white bars), MPSIIIB mice (1, red bars), MPSIIIB×TLR4^−/−^ mice (2, yellow bars), MPSIIIB×MyD88^−/−^ mice (3, green bars), or MPSIIIB mice in which the genetic defect was corrected in the brain by a single intracerebral injection of AAV2.5-hNaGlu vector (4, blue bars) were analyzed at the age of 8 months. Inflammation markers were studied in cortical samples stained with anti-CD11b antibody (green in A) and by measuring the relative amounts of MIP1α (C) and IL1ß (D) mRNAs by quantitative RT-PCR. Disease markers were studied in cortical samples stained with the anti-ScMAS antibody (green in B), and by measuring the relative amounts of GAP43 mRNAs (E), the accumulation of GM2/GM3 gangliosides (F), and the frequencies of vacuolated neurons or astrocytes (G) and vacuolated microglia (H). Immunofluorescence (A and B): nuclei are stained in blue with Hoescht, scale bars: 20 µm for CD11b, 50 µm for ScMAS. Representative pictures from 3 MPSIIIB, 3 MPSIIIB×TLR4^−/−^ and 3 MPSIIIB×MyD88^−/−^ mice. RT-Q-PCR (C, D, E): mRNA amounts are expressed relative to the reference ARPO mRNA [53]. Pathology (G and H): semi-thin sections (1 µm) were stained with toluidin blue. At least 90 neurons/astrocytes were scored per mm^2^ section surface. Values are from 3 MPSIIIB, 3 MPSIIIB×TLR4^−/−^ and 3 MPSIIIB×MyD88^−/−^ examined mice. Examples of the morphology of cells that were scored as normal neurons or astrocytes, or vacuolated neurons or astrocytes are illustrated in figure S4. Asterisks indicate significant difference with wild type mice and crosses indicate significant differences with untreated MPSIIIB mice (p<0.05, Mann and Whitney non-parametric test). Number of mice used for GAG, gangliosides and mRNA analyses: wild type mice, n = 5; MPSIIIB mice, n = 10; MPSIIIB×TLR4^−/−^ mice, n = 4; MPSIIIB×MyD88^−/−^ mice, n = 1; MPSIIIB+AAV2.5-hNaGlu mice, n = 3.

Eight-months-old MPSIIIB mice showed severe inflammation in the brain (figure 5A,C,D). CD11b staining was intense and associated to astrocytosis, as shown by increased reactivity for glial fibrillary acidic protein (GFAP), a marker of astrocytes (astrocytosis was not detected at 10 days and was very mild at 3 months, figure S3). Amounts of IL1ß and MIP1α mRNAs were high. Although slightly milder than in MPSIIIB mice, inflammation was also intense in the cortex of doubly mutant mice (figure 5A,C,D), suggesting it was mostly independent of TLR4 and MyD88 at this stage, in contrast to the observations made at 3 months. However, the disappearance (IL1ß and MIP1α, figure 5 C,D) or the reduction (CD11b, figure 5A) of inflammatory markers in treated MPSIIIB mice (figure 5A,C,D) indicated that the inflammatory reaction was nevertheless triggered by HS oligosaccharides, although presumably through different mechanisms in aged mice than in younger animals.

As expected, disease markers were more severely affected in MPSIIIB mice at 8 months than at 3 months (figure 5B and E–H): GM2 and GM3 ganglioside levels were higher, cell vacuolation was more prominent and ScMAS immunostaining was more intense. GAP43 mRNA levels, which decrease with age in wild type mice, at the opposite increased in MPSIIIB mice, switching from abnormally low levels in young mice to abnormally high levels in aged mice, as previously described [30]. Similar features were observed in doubly mutant mice (figure 5B and E–H), whereas 8-months-old mice that received gene therapy were similar to age-matched wild type mice (figure 5B and E–H). This observation confirmed that the toxic effects of HS oligosaccharides on the brain of ageing mice were mostly independent of microglial cell priming through TLR4 and MyD88.

## Discussion

In MPSIII, neuroinflammation and neuropathology are caused by the production and accumulation of HS oligosaccharides that are absent in normal cells. The aim of the present study was to examine causative links between HS oligosaccharide accumulation, inflammation and disease markers in the cortex of MPSIIIB mice. Our approach associated microglial cell cultures, modulation of brain inflammation in cross-bred mice and disease correction by gene therapy. Results provided evidence that HS oligosaccharides primed microglial activation through TLR4 and MyD88 both in vitro and in the MPSIIIB mouse brain. However, suppression of microglial priming in MPSIIIB mice deleted for TLR4 and MyD88 showed dissociation between inflammation, which was absent or drastically reduced in young animals (10 days and 3 months), and expression of disease markers, which was unchanged. We conclude that neurodegeneration progresses independently of the stimulation of the innate response by HS. Nevertheless, injuries subsequent to the degenerative process progressively created a chronic inflammatory state in the brain of ageing MPSIIIB mice (8 months), which was independent of TLR4 and MyD88, and which in turn may have exacerbated pathology. Injuries that possibly stimulated chronic inflammation in ageing MPSIIIB mice are the alteration of the blood-brain barrier, as documented in mice with GM1 or GM2 gangliosidosis [20], the secondary accumulation of GM2 and GM3 ganglisosides [33] and the cell death.

Microglial activation by HS oligosaccharides is consistent with a previous report showing that these molecules activate dendritic cells through TLR4 [34]. Our experimental controls indicated that only HS oligosaccharides, and not non-fragmented HS or chondroitin sulfate activated microglia in vitro. HS oligosaccharide fragments are produced in early endosomes by heparinases and thereafter transferred to lysosomes for further digestion by exoglycanases. The size of abnormal undigested oligosaccharides accumulating in exoglycanase deficient cells, such as oligosaccharides with terminal α-linked N-acetylglucosamine in MPSIIIB, ranges from single to multiple repeats of disaccharides. Further studies will specify the structure, sulfation and acetylation of HS oligosaccharide species responsible for priming microglial activation.

In vitro assays showed a moderate effect of HS oligosaccharides with respect to TNFα mRNA production and a much more robust effect on MIP1α and IL1β mRNA expression. We therefore focused in vivo studies on the latter two factors. They are principally produced in the brain by activated microglia and/or astrocytes. In 10-days-old MPSIIIB mice, we observed increased CD11b staining and normal GFAP staining, suggesting that MIP1α and IL1β mRNAs were produced by activated microglia. Although GAG concentration in brain extracts was still in the normal range at this stage, abnormal HS oligosaccharides were already produced because of the enzyme defect, and therefore possibly triggered microglial cell priming. Other stimuli that could trigger microglial cell priming, like cell vacuolation and death and/or secondary accumulation of GM2 and GM3 gangliosides [35], were absent at this stage. Consistently with the stimulation of microglial cell priming by HS oligosaccharides in the brain, deletion of the innate immune response mediators TLR4 or MyD88 in MPSIIIB mice showed substantial reduction of CD11b staining and of the expression level of IL1β and MIP1α mRNAs at 10 days and 3 months, as observed in vitro.

Early MIP1α production in the MPSIIIB mouse brain is reminiscent of the implication of this chemokine in the neurodegenerative process associated with Sandhoff disease [36]. Possibly, oligosaccharides with terminal ß-linked N-acetylglucosamine could prime microglia in this disease, as suggested [37]. Microglial activation and increased MIP1α gene expression were also observed in the mouse models of GM1 and GM2 gangliosidoses before the onset of neurodegeneration [20].

An important observation in doubly mutant mice versus MPSIIIB mice was the dissociation at 10 days, and more clearly at 3 months, between the reduced expression of inflammation markers and the severe expression of disease markers. Less intense CD11b staining and reduced amounts of MIP1α and IL1ß mRNAs were not associated with delayed occurrence of cell vacuolation, ganglioside accumulation, detection of ScMAS or altered expression of GAP43 mRNAs, which are relevant markers of the progression of the neurodegenerative process in MPSs [26], [29], [30], [38]. Other markers were not investigated in doubly mutant mice. They include brain cell apoptosis and Purkinje cell loss, which were not consistently observed in MPSIIIB mice [25], [30], [39]. The study of behavioral manifestations, which led to controversial conclusions in MPSIIIB mice [28], [31], [39], and that of the mean life-span were not performed because of the limited number of available doubly mutant mice. Investigations of these markers in Sandhoff mice showed delayed occurrence when affected animals did not express MIP1α [36]. It remains to be established whether the apparently different impact of priming of the innate immune response in Sandhoff and MPSIIIB in mice is due to difference in the courses of these diseases in mice, difference in the mechanisms leading to brain inflammation, difference in the invalidated innate immune response genes, or difference in the investigated disease markers.

In MPSIIIB mice that received AAV vector mediated gene therapy directed to the brain, microglial activation had been primed by HS oligosaccharides before treatment. The clearance of HS oligosaccharides after treatment was associated with the absence of expression of disease markers and the very low, though often still detectable expression of some inflammation markers, including slightly increased CD11b signals and IL1ß mRNA amounts. It is conceivable that trace amounts of residual HS oligosaccharides production at locations in the brain where enzyme delivery was less efficient led to persisting chronic stimulation of the innate immune response. As a consequence, these mice may be more susceptible to additional stimulation, as proposed for prion disease in which priming of microglial activation by Prp^sc^ exacerbated innate immune responses to microbial pathogens [13]. This hypothesis is supported by the observation that microglial cells isolated from MPSIIIB mice produced higher amounts of MIP1α and IL1ß mRNAs in vitro in response to LPS or HS oligosaccharides than wild type mouse microglia. A further investigation of this issue in MPSIIIB mice is worthwhile, since it may be relevant to the outcome of treatments in children.

Our results indicate that inflammation may have different causes and consequences depending on disease progression in the brain of MPSIIIB mice. Priming of microglial activation by HS oligosaccharides occurs at very early stage of the disease, whereas pathology is still very mild. Activated microglia are thought to act as effector cells in the degeneration of neural cells in the central nervous system. Soluble factors released by activated microglia are capable of inducing intracellular swelled vesicles (beads) in neuron dendrites and axons [40], [41]. However, although this mechanism might be relevant to the development of vacuolation and ScMAS accumulation in MPSIIIB neuron, it is presumably not involved in MPSIIIB since the appearance of these disease markers was not delayed when microglial activation by HS oligosaccharides was reduced in young doubly mutant mice. It is therefore likely that at early stage of the disease pathology developing in the MPSIIIB central nervous system is cell-autonomous rather than environmental. Observation made in 8-months-old MPSIIIB and doubly mutant mice suggest that the situation may be different at later stage of the disease, when additional stimuli of innate immunity subsequent to pathology development are likely predominant. Responses to these stimuli is independent of TLR4 and MyD88 but may be exacerbated by the permanent stimulation of microglia by HS oligosaccharides. They presumably induce a self-sustained chronic inflammation that is potentially harmful to the central nervous system.

## Materials and Methods

### Reagents

Media, fetal calf serum and antibiotics were from Invitrogen (Carlsbad, California). Lipopolysaccharide (LPS, from Escherichia coli 0111∶B4), bovine HS (Heparin monosulfate sodium-potassium salt), desulfated heparin (partially acetylated; semi-synthetic), Polymyxin B, E-Toxate kit (*Limulus* Amebocyte Lysate, LAL) were from Sigma (St Louis, MO, USA). Polyinosine-polycytidylic acid (poly[I∶C]) was from InvivoGen (Toulouse, France).

### Patients, urine collection, HS oligosaccharide purification, microglia cultures

- Patient 1 (11-years-old, GAG1 and HS1) and patient 2 (8-years-old, GAG2 and HS2) were diagnosed for MPSIIIB before the age of 5 years. Both had severe neurological manifestations at the time of urine collection. Urines were collected under sterile conditions and immediately stored at −20°C. According to the French law (Code de la Santé Publique, art. L1121-1), urine collection without diagnostic or prospective investigation purpose is not considered as Biomedical Research and therefore does not require ethical committee approval.

- Isolation and characterization of HS oligosaccharides from patient urine. GAGs were isolated as previously described [42]. Urines were acidified to pH 5.0–6.0 with acetic acid and centrifuged. Cetylpyridinium chloride (CPC, 200 µL 5% w/v) was added to supernatants for 12 hours at 4°C and centrifuged. Pellets were successively washed with ethanol saturated with sodium chloride, ethanol and ether, dried and resuspended in 0.6 mol/L NaCl at 4°C for 3 hours. After centrifugation, supernatants with solubilized GAGs were precipitated with ethanol at 4°C for 12 hours. Pellets were washed as before, resuspended in water and used as GAG fraction.

- Isolation of HS from the GAG fraction was performed as previously described [43]. GAG pellets were dissolved in 0.25 M NaCl and applied on a Dowex 1×2 column (100–200 mesh, Cl^−^ form) previously equilibrated with 0.25 M NaCl. After washing with 0.25 M NaCl, stepwise elution of HS was performed with 0.5 M, 0.75 M, 1 M and 1.25 M NaCl. Fractions were assayed for uronic acid content by hexuronic acid measurement [42] and analyzed by cellulose acetate electrophoresis. Fractions containing HS were pooled and precipitated with 4 volumes of ethanol at 4°C for 12 h. After centrifugation, pellets were dried, resuspended in water and used as HS fraction. Preparations used for microglia activation assays were free of detectable endotoxin contaminant, as shown by *Limulus* Amebocyte Lysate (LAL) assays. Oligosaccharides in HS fractions were derivatised with 1-phenyl-3-methyl-5-pyrazolone and analysed by ESI-MS/MS using a PE Sciex API 3000 triple quadrupole mass spectrometer as described previously [32].

- Microglia cultures. Three day-old C57BL/6J mice were obtained from Janvier Inc. (Le Genest-St-Isle, France). TLR4 deficient [44] and MyD88 deficient [45] C57BL/6J mice were obtained from the laboratory of Shizuo Akira, backcrossed eight times to the C57Bl/6 background and bred in the central animal facility of the Pasteur Institute under SPF conditions. Primary murine microglia cultures were prepared as previously described [46]. Briefly, cerebral cortices without meninges were mechanically and enzymatically dissociated with proteinase (10 U/mL) and DNase (4000 U/mL). After adding of DMEM supplemented with 10% endotoxin free heat-inactivated FBS and 50 µg/mL gentamycin, cells were plated in DMEM 10% FCS on 100 mm Petri dishes coated with 1.5 µg/mL poly-DL-ornithine (3 to 15 kDa) at a density of 3×10^6^ cells/dishes. Mixed glial cultures were maintained at 37°C with 5% CO_2_ and medium was changed after 4 days. After 12 days, dishes were shaken at 150 rpm for 90 min to detach non-adherent microglia. Detached cells were collected and plated on either 24-well tissue culture plate slides or in 6-well tissue plates at a density of 67,500 cells per cm^2^ and maintained at 37°C for 1 hour. After one hour at 37°C, loosely adherent contaminating oligodendrocytes were removed from the cultures. Adherent microglia was maintained at 37°C with 5% CO_2_ for one day before treatment. Culture supernatants or total RNAs were harvested at the indicated time after treatment and stored at −80°C until assayed. Polymyxin B (10 µg/mL) was added to cultures one hour before treatment with inducer.

- Immuofluorescence assays on microglia cultures. Antibodies: mouse anti-rat ED1 monoclonal antibody (mAb, 1∶200, clone OBT1150, Immunological direct), rat anti-mouse CD11b mAb (1∶500, clone M1/70, BD Biosciences). Microglia seeded onto 15 µg/mL poly-DL-ornithine round coverslips was washed in PBS and fixed with 4% paraformaldehyde for 15 minutes. After 3 washes with PBS, cells were incubated overnight at 4°C with primary antibody in PBS, 1% bovine serum albumine (BSA), 2% normal goat serum (anti-CD11b) or in PBS, 1% BSA, 2% normal goat serum, 0.3% Triton (anti-ED1, anti-CD68, anti-GFAP). Bound antibodies were revealed by one-hour incubation at room temperature with Alexa Fluor 488 or 545-conjugated antibodies (1∶500, Invitrogen) and 1 µg/mL DAPI. Coverslips were mounted in Fluoromount-G (Southern Biotech, Birmingham, AL). Observations were done on Axioplan 2 imaging optic microscope (Zeiss, Le Pecq, France).

- Mouse TNF-α ELISA was from Biosource International (Camarillo, CA, USA). Sensitivity was 10 pg/mL.

### Mice, gene transfer to the brain, tissue processing and analysis

- Mouse experiments were approved by the Institut Pasteur ethical committee for animal research and performed by authorized investigators (authorization no. 75–268, Ministère de l'Agriculture et de la Pêche). C57Bl/6^NaGlu+/−^ MPSIIIB mice were obtained from Pr. E. Neufeld (UCLA, Los Angeles, CA) [28]. C57Bl/6^TLR4−/−^ [44] and C57Bl/6^MyD88−/−^ [45] mice were obtained from the laboratory of Shizuo Akira. C57Bl/6^NaGlu−/−,TLR4−/−^ and C57Bl/6^NaGlu−/−,MyD88−/−^ were generated by breeding C57Bl/6^NaGlu+/−,TLR4+/−^ or C57Bl/6^NaGlu+/−,MyD88+/−^, respectively. Doubly mutant mice were identified by genotyping. They were sterile and could not be propagated. Absence of NaGlu, TLR4 or MyD88 expression was verified in brain extracts (table S1). Correction of the genetic defect and phenotype in the brain was performed as described [31], using AAV vectors coding for the missing enzyme NaGlu.

- Selection of single and doubly mutant mice. Genotype at NaGlu, TLR4 and MyD88 loci was determined by PCR, using tail DNA. Primers used for the detection of wild type alleles: α-N-acetylglucosamine gene exon 6: forward: 5′-TGGTCAGCCTGTGCTATGAG-3′; reverse: 5′-AGGTACCCAGCAAGAAGTGG-3′. TLR4 gene exon 3: forward: 5′-TGTTGCCCTTCAGTCACAGAGACTCTG-3′; reverse: 5′-CGTGTAAACCAGCCAGGTTTTGAAGGC-3′. MyD88 gene exon 4: forward: 5′-AGCCTCTACACCCTTCTCTTCTCCACA-3′; reverse: 5′-AGACAGGCTGAGTGCAAACTTGTGCTG-3′. Primers used for the detection of mutated alleles: α-N-acetylglucosamine gene exon 6: forward: 5′-GGAGAGGCTATTCGGCTATGACTG-3′, reverse: 5′-GGACAGGTCGGTCTTGACAAAAAG-3′; TLR4 gene exon 3: forward: 5′-TGTTGCCCTTCAGTCACAGAGACTCTG-3′, reverse: 5′-ATCGCCTTCTATCGCCTTCTTGACGAG-3′; MyD88 gene exon 4: Forward: 5′-AGCCTCTACACCCTTCTCTTCTCCACA-3′, reverse: 5′-ATCGCCTTCTATCGCCTTCTTGACGAG-3′

- Breeding procedures. MPSIIIB (NaGlu^−/−^) were produced by crossing NaGlu^+/−^ mice and selection of the progeny on genotype. Breeding of NaGlu^−/−^×NaGlu^−/−^ was possible, though poorly efficient. NaGlu^−/−^×TLR4^−/−^ and NaGlu^−/−^×MyD88^−/−^ were produced by selection of the progeny of cross-breeding between NaGlu^−/−^ and TLR4^−/−^ or NaGlu^−/−^ and Myd88^−/−^. Doubly mutant mice were sterile. All experiments involving doubly mutant mice were therefore performed on F1 mice. The absence of NaGlu activity was verified by enzyme assay in brain extracts. The absence of detection of TLR4 or MyD88 was verified by quantitative RT-PCR in brain extracts and by western blot in liver extracts (table S1).- Adeno-associated virus vector and vector production. The AAV2.5-hNaGlu vector is a hybrid AAV vector in which the capsid is from the AAV5 serotype and the genome from the AAV2 serotype. The vector genome contains the Inverted Terminal Repeats (ITRs) of AAV2, the mouse phosphoglycerate kinase-1 gene promotor (mPGK), the human NaGlu cDNA [47] and the BGH polyadenylation sequence. A control AAV vector was similarly constructed except that mPGK promoter sequences were deleted. Vector batches were produced by transient transfection of HEK293 cells and purified using two successive CsCl gradients. Purified fractions were pooled and dialysed against PBS/Ca^2+^/Mg^2+^ (Biowhitaker), as described [48]. Vector genomes (vg) were quantified by dot blot.

- Intracranial injections. Six-week-old MPSIIIB^−/−^ mice were anesthetized with ketamine/xylazine (0.1/0.01 mg/g body weight) and installed on a stereotactic frame (David Kopf Instruments, Tujunga, CA) (n = 9). The skull was exposed by a small incision. Five microliters of the AAV2.5-hNaGlu vector or the control AAV vector (3×10^9^ vg) were injected into the striatum with a Hamilton syringe with a 30-gauge blunt tip needle mounted to the stereotactic frame (coordonates +0.7 mm anterior to bregma, 2 mm left lateral to the midline and 4.5 mm depth). 2.5 µl of vector was delivered with an ultramicropump (World Precision Instruments, Hertfordshire, UK) at a rate of 500 nl/min. The needle was then brought up 0.5 mm and the rest of the volume was injected at the same rate. The needle was slowly withdrawn, the scalp was closed and the animals were returned to recovery cages.

- Brain tissue processing. *Mice that did not receive AAV vector injection*: biochemistry, RT-PCR and imaging studies were all performed in the same mouse. After perfusion with PBS, brains were removed and hemispheres were separated. The right hemisphere was collected for GAG assay. The left hemisphere was sliced. Cortical fragments (3 mm^3^) were collected for RT-PCR analysis, immunofluorescence analysis and semi-thin sections. Fragments for RT-PCR were immediately frozen in nitrogen and stored at −135°C until RNA extraction. Fragments for immunofluorescence were fixed with 4% paraformaldehyde (PFA) for 1 hour at +4°C, rinsed, immersed in 30% sucrose and embedded in O.C.T. (Tissue-tek, Sakura) and stored at −80°C until cryosection. Fragments for semi-thin sections were fixed by immersion with 3.6% glutaraldehyde at +4°C, post-fixed in 2% aqueous osmium tetroxide and embedded in epon-araldite. *Mice that received AAV vector injection*: treated MPSIIIB mice received the AAV2.5-hNaGlu vector. These mice were used for either biochemical or pathology analyses. Control normal mice received the control AAV vector. These mice were used for biochemical analyses, providing information about inflammatory response to AAV vector particle injection in the brain. Biochemical analyses were performed using cortical fragments collected from the injected hemisphere and immediately frozen in nitrogen for RT-QT-PCR analysis. They included enzyme activities and GAG assay. Pathology analyses were performed after intracardial perfusion with 4% PFA. Brains were removed, hemispheres were separated and cortical fragments were collected. They were used for immunofluorescence and semi-thin sections, as described below.

- Semi-thin sections. One-micrometer-thick sections were prepared, stained with toluidine blue for 2 hours and examined with a bright field microscope (Axiovision software, Carl Zeiss, MicroImaging, Inc.). Images were acquired with a 100× objective lens from 5 randomly chosen areas of the cerebral cortex.

- Immunofluorescence on brain sections. Ten µm coronal cortex cryosections were incubated overnight at +4°C with biotin-conjugated rat anti-mouse CD11b monoclonal antibody (1∶200; clone no. M1/70, BD Biosciences), rabbit anti-cow GFAP (1∶500, Dako) or anti-subunit c of mitochondrial ATP synthase (ScMAS) rabbit serum (1∶1000, a gift Pr. D. Palmer, Lincoln University, Canterbury, New-Zeland). After pre-incubation in blocking buffer (10% normal goat serum, 10% normal donkey serum in PBS for anti-CD11b and anti-GFAP; 2% normal goat serum, 5% bovine serum albumin, 0.02% saponin for anti-ScMAS) for 30 min at room temperature, bound antibodies were revealed with streptavidin Alexa Fluor® 488 (1∶1000, Molecular Probes, anti CD11b), or goat anti-rabbit Alexa Fluor® 647 (1∶500, Invitrogen, anti-GFAP and anti-ScMAS) for 1 hour at room temperature. Nuclei were stained with Hoechst (1∶500, Sigma), sections were mounted with Fluoromount (Southern Biotech) and examined by confocal fluorescence microscopy (CD11b, 63×, LSM510, Carl Zeiss, MicroImaging, Inc.) or fluorescence microscopy (GFAP, 20×, Axiovision software, Carl Zeiss, MicroImaging, Inc.). Images were acquired from 3 to 6 randomly chosen fields.

- Lysosomal enzyme assays on brain sections. Anesthetized animals were perfused with PBS and cerebral hemispheres were separated. Tissue was homogenised in water, submitted to 10 freeze/thaw cycles and clarified by centrifugation at 13,000 rpm for 3 min. NaGlu and total ß-hexosaminidases activities were determined by fluorometric assays using 4 mM 4-methylumbelliferyl-N-acetyl-α-D-glucosaminide (Calbiochem) for 2 hours [49] or 1 mM 4-methylumbelliferyl-2acetamido2deoxy-α-D-glucopyranoside (Sigma) substrate for 5 minutes [50], respectively. One catalytic unit corresponds to the hydrolysis of 1 nmol of substrate per hour and expressed per mg of proteins in extracts. Protein concentration was determined with a BCA protein assay kit with bovine serum albumin as the standard.

- Analysis of glycosaminoglycans in brain extracts. Frozen samples were homogenized with a minimum volume of water (10% vol/weight). Defatted pellets were dried and weighed. Dried residues were digested overnight at 65°C with papain (0.3% w∶v) in 3 ml of 100 mM sodium acetate buffer pH 5.5 containing 5 mM cysteine and 5 mM EDTA. After centrifugation, GAGs were measured in the supernatant with a dimethylmethylene blue dye binding assay. Briefly, 200 µl of the supernatant was added to 2.5 ml of dimethylmethylene blue reagent [51] and the absorbance at 535 nm was measured. Heparan sulfate was used as standard. Data (means of duplicates) were expressed as µg of GAGs per mg of dried pellet.

- Ganglioside assay. The analysis of gangliosides was performed as has been previously described [52]. Data (mean of duplicates) were expressed as mole percentage of total gangliosides.

### Quantitative RT-PCR

- Procedure. Total RNA was extracted using Trizol (Invitrogen) from approximately 300,000 cells or from 50 mg of cortex and suspended in 30 µL of water. One microgram of total RNA was used to synthesize cDNA with oligo (dT)_12–18_ (200 ng, Roche Applies Science, Meylan, France) and M-MLV reverse transcriptase (Superscript III, Invitrogen). Quantitative PCR was performed in a Model 7000 Sequence Detector (Applied Biosystems, Foster City, CA) with 100 ng of cDNA and the SYBR Green PCR Master Mix (according to Applied Biosystems procedures). Amplification parameters: 50°C for 2 minutes, 95°C for 10 minutes, 95°C for 15 seconds, 60°C for 1 minute. Each sample was analyzed in triplicate. Negative controls included omission of reverse transcriptase at the cDNA synthesis step and omission of the template at the PCR step. Additional controls performed for each cDNA amplification included optimization of primer concentration, assessment of amplification efficiency and detection of possible primer dimerization through analysis of dissociation curves. Ct (Cycle threshold) values were determined as the numbers of PCR cycles at which specific amplification of the target sequence occurred. Ct superior to 38 were considered as background signal. cDNA amounts were expressed as 2exp(Ct1–Ct2), in which Ct1 is a reference Ct measured for the amplification of ARPO (Acidic ribosomal phosphoprotein) cDNAs [53] and Ct2 is the Ct measured for the amplification of the examined cDNA. Samples were analyzed in triplicate.

- Primers: ARPO, forward: 5′-TCCAGAGGCACCATTGAAATT-3′, reverse: 5′-TCGCTGGCTCCCACCTT-3′; TNF-α, forward: 5′-GAGTGACAAGCCTGTAGCCCA-3′, reverse: 5′-GCGCTGGCTCAGCCAC-3′; IL-1ß, forward: 5′-CAACCAACAAGTGATATTCTCCATG-3′ reverse: 5′-GATCCACACTCTCCAGCTGCA-3′; MIP1α, forward: 5′-CTGCAACCAAGTCTTCTCA-3′, reverse: 5′-GCATTCAGTTCCAGGTCAGT-3′; GAP43, forward: 5′-CACCATGCTGTGCTGTATGAGA-3′, reverse: 5′-TGTTCAATCTTTTGGTCCCTCATCA-3′.

## Supporting Information

Table S1 (0.03 MB DOC)

Figure S1 (0.94 MB DOC)

Figure S2 (0.36 MB DOC)

Figure S3 (0.23 MB DOC)

Figure S4 (1.57 MB DOC)
