# Supplementary material for: Early Neurodegeneration Progresses Independently of Microglial Activation by Heparan Sulfate in the Brain of Mucopolysaccharidosis IIIB Mice
Source: PLoS One. 2008 May 28;3(5):e2296. doi: 10.1371/journal.pone.0002296 (PMC2396504; doi:10.1371/journal.pone.0002296)
Supplement: Table S1 — (0.03 MB DOC) [file pone.0002296.s001.doc]

*Supporting table 1*. **Absence of detection of TLR4 and MyD88 in doubly mutant mice**

Total RNA was extracted from 10 mg of cortex collected from wild type, MPSIIIB, MPSIIIB/TLR4^-/-^ and MPSIIIB/MyD88^-/-^ mice. RNA (1 µg) was reverse transcribed and amounts of cDNA coding for TLR4 and MyD88 or the reference protein ARPO were measured by Q-PCR. Indicated values are means ± SEM of ratios of TLR4 and MyD88 mRNAs to ARPO measured in two independent experiments.

Primers:

-TLR4:

Forward: 5’-TCTGATCATGGCACTGTTCTTCTC-3’

Reverse: 5’-TCTGATCCATGCATTGGTAGGT-3’

-MyD88:

Forward: 5’-TGTCCCACAAACAAAGGAACTG-3’

Reverse: 5’CAGTAGCAGATAAAGGCATCGAAA-3’

| Phenotype | TLR4 | MyD88 |
| --- | --- | --- |
| Wild Type | 0.07±0 | 0.045±0.001 |
| MPSIIIB | 0.052±0.005 | 0.049±0.004 |
| MPSIIIB/TLR4-/- | < 10^-5^ | 0.021±0.01 |
| MPSIIIB/MyD88-/- | 0.078±0.012 | < 10^-5^ |
