# Supplementary material for: Early Neurodegeneration Progresses Independently of Microglial Activation by Heparan Sulfate in the Brain of Mucopolysaccharidosis IIIB Mice
Source: PLoS One. 2008 May 28;3(5):e2296. doi: 10.1371/journal.pone.0002296 (PMC2396504; doi:10.1371/journal.pone.0002296)
Supplement: Figure S1 — (0.94 MB DOC) [file pone.0002296.s002.doc]

*Figure S1.*  **Electrophoretic analysis of GAGs and HS**

GAGs isolated from urines of MPSIIIB patients (lanes 1 and 2) or healthy control (ctrl, lane 3), and HS purified from urine of patient 1 (lane 4) were analyzed on cellulose acetate plates saturated with barium acetate and stained with 0.02% dimethylbenzene in 1% acetic acid. Sharp signals (lanes 1, 2 and 4) correspond to HS, and thinner signals below (lanes 1, 2 and 3) to chondroitine sulfate (CS).
