# Supplementary material for: Early Neurodegeneration Progresses Independently of Microglial Activation by Heparan Sulfate in the Brain of Mucopolysaccharidosis IIIB Mice
Source: PLoS One. 2008 May 28;3(5):e2296. doi: 10.1371/journal.pone.0002296 (PMC2396504; doi:10.1371/journal.pone.0002296)
Supplement: Figure S2 — (0.36 MB DOC) [file pone.0002296.s003.doc]

*Figure S2.* **Immunofluorescence analysis of microglial activation**

Microglial cells were seeded onto poly-DL-ornithine coated glass coverslips and either untreated (mock), or exposed for 6 hours to 5µg/mL desulfated heparin (Heparin), 5 µg/mL bovine HS (Bovine HS), 5 µg/mL GAGs isolated from urines of a healthy individual (normal GAG), 1 µg/mL LPS (LPS), 5 µg/mL HS from patient 1 (HS1), or 5 µg/mL HS from patient 2 (HS2). After fixation with 4% paraformaldehyde, cells were labeled with anti-ED1 (left column, in green) and anti-CD11b (middle column, in red) antibodies and nuclei were stained with DAPI (right column, in blue). The same field is shown in the 3 columns. Scale bar, 10 µm.


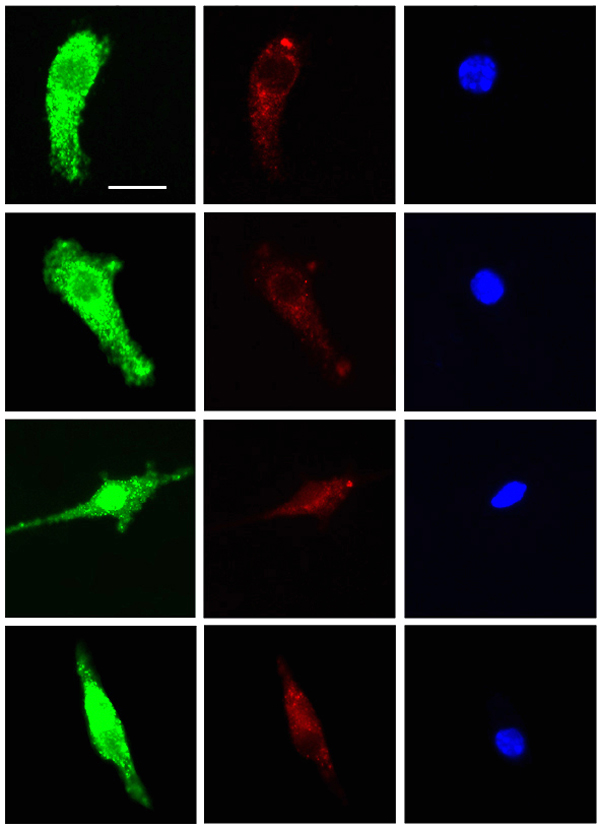
 **ED1 CD11b DAPI**

**Mock**

**Heparin**

**Bovine HS**

**GAG from**

**normal**

**urines**

**
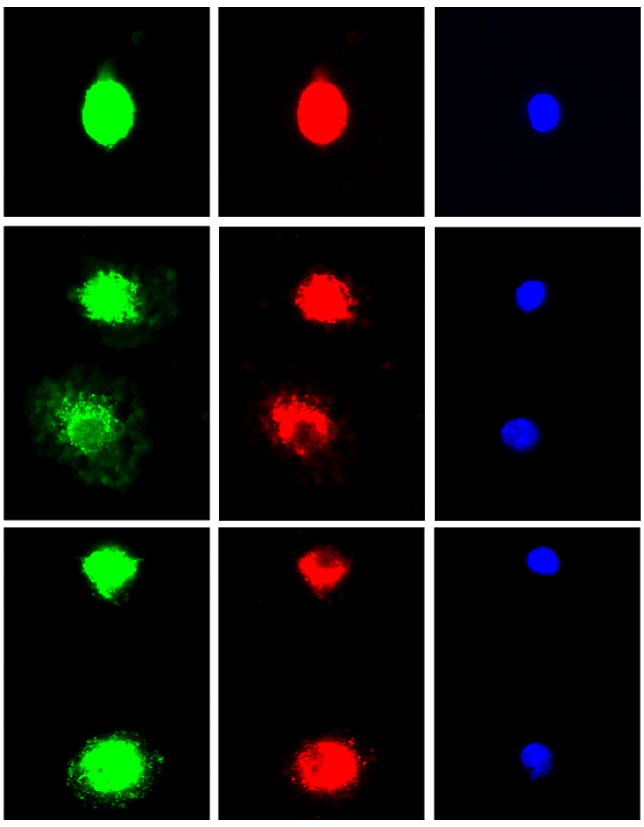
**

**LPS**

**HS1**

**HS2**
