# Supplementary material for: Early Neurodegeneration Progresses Independently of Microglial Activation by Heparan Sulfate in the Brain of Mucopolysaccharidosis IIIB Mice
Source: PLoS One. 2008 May 28;3(5):e2296. doi: 10.1371/journal.pone.0002296 (PMC2396504; doi:10.1371/journal.pone.0002296)

*Figure S3*. **Astrocytosis in mouse cortex**

Pictures show examples of subcortical cryosections stained with anti-GFAP antibody (white) and

Hoescht (blue nuclei). Wild type (a-c), MPSIIIB (d-f), MPSIIIBxTLR4-/- (g-i), MPSIIIBxMyD88-/- (j-l) mice or MPSIIIB mice treated by a single intracerebral injection of AAV2.5-hNaGlu vector (m and n) were killed at the age of 10 days (upper row), 3 months (6 weeks after vector injection for treated MPSIIIB, middle row) or 8 months (26 weeks after vector injection for treated MPSIIIB, bottom row). Scale bar, 50µm.


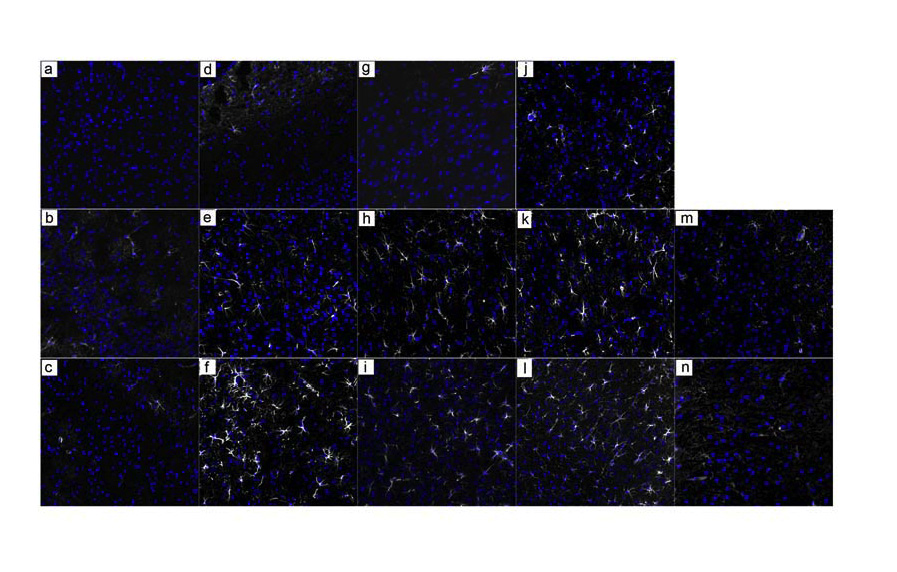

Supplement: Figure S3 — (0.23 MB DOC) [file pone.0002296.s004.doc]
