# Supplementary material for: Early Neurodegeneration Progresses Independently of Microglial Activation by Heparan Sulfate in the Brain of Mucopolysaccharidosis IIIB Mice
Source: PLoS One. 2008 May 28;3(5):e2296. doi: 10.1371/journal.pone.0002296 (PMC2396504; doi:10.1371/journal.pone.0002296)
Supplement: Figure S4 — (1.57 MB DOC) [file pone.0002296.s005.doc]

*Figure S4.* **Intracytoplasmic vacuolation in MPSIIIB mouse brain cells**

The two top rows show the morphology of cells scored as microglia (arrows in a and b) or as neuronal or astrocytic cells (arrows in c and d) in 8-months-old wild type mice (a and c) or MPSIIIB mice (b and d). Scored numbers are shown in figures 4G,H and 5G,H. Microglia apposed to cells that were presumably neurons are visible in a and b (arrow heads). MPSIIIB microglia contains enlarged empty vacuoles (b). Large cells with dark cytoplasm and dark nucleus, which are presumably neurons, and which contain numerous small clear vacuoles in MPSIIIB, were scored (d). Large cells with clear cytoplasm and clear nucleus (arrow heads in c, d), which are presumably neurons but in which vacuolation is hardly visible, were not scored. Examples of normal and pathological perivascular (e, f) and meningeal (g, h) tissues are shown in the two bottom rows. Vacuolated endothelial cells (arrows in f) and vacuolated cells that were presumably pericytes (arrow heads in f) are visible. Arrows in h indicate meningeal cells filled up with clear vacuoles. Semi-thin sections (1 µm thick) were stained with toluidine blue. Scale bars, 10 µm.
